# Highlights from the first *e*cancer–Liga Colombiana contra el Cancer conference, 17–18 November 2016, Bogota, Colombia

**DOI:** 10.3332/ecancer.2017.730

**Published:** 2017-03-28

**Authors:** Carlos Castro

**Affiliations:** Liga Colombiana contra el Cancer, Bogota, Colombia

**Keywords:** geriatric oncology, vaccination against HPV, gastric cancer, multiple myeloma, thrombosis

## Abstract

The first oncology conference organised by *e*cancer and the Liga Colombiana contra el Cancer took place on 17–18 November 2016 in Bogota. It was a highly successful event owing to the number of participants, the quality of the speakers, and the academic programme. Around 48 professors from 8 different countries came and shared their knowledge and experience of cancer management. They also talked about the most recent developments noted or achieved in this area. The keynote speech from Dr Nubia Muñoz was of great interest which was related to the safety of a HPV vaccine and the implications of a mass vaccination programme in developing countries. Geriatric oncology and palliative care were also topics that sparked great interest during the event.

The First Oncology Congress organised by *e*cancer in association with the Liga Colombiana contra el Cancer took place on 17 and 18 November 2016. It was a success owing to the amount of attendees (390) it constituted. They were radiotherapists, clinical oncologists, oncologist nurses, gynaecologists, haematologists, urologists, palliative care professionals, and medical students who welcomed the high quality guest speakers from Spain, France, USA, Chile, Mexico, Peru, Argentina, Cuba and Colombia.

It was a heartfelt tribute to the co-founder of *e*cancer, Professor Umberto Veronesi, who recently passed away and left a large void in the field of global oncology. His medical, personal, and leadership qualities were admired by all in his lifetime. Undoubtedly, he was a great man who will be remembered and admired for many years to come.

During the congress, the ‘Umberto Veronesi Award’, established by the man himself was given to Colombian oncologist Dr Haroldo Estrada for his work using film as a tool for communication and teaching. This was also in recognition of his services to both teaching and research in the city of Cartagena de Indias.

The congress focused on topics that were considered to be of great importance for Colombia and Latin America in general [1]. Therefore, Dr Carlos Castro raised the topic of the problem of cancer in the elderly population and the dearth of geriatric oncologists. He said this group of patients is ever increasing and cancer is a very common illness among them. It is calculated that approximately 40% of all malignant tumours are diagnosed in people over the age of 65. Social, financial, and emotional implications were widely discussed and a call was made for the new generation of oncologists to become involved in the area and find a way to involve oncologists in geriatric training [2]. Professor William Otero, Chairman of Gastroenterology at the Universidad Nacional, who has extensive experience in teaching and research has skilfully developed a module on gastrointestinal cancer. It focused on the role of Helicobacter pylori and salt content in diet as predisposing factors in the genesis of gastric cancer. He made a call for doctors to solicit digestive endoscopy for patients that showed signs of dyspepsia. This is due to the fact that gastric cancer is one of the most dangerous cancers in Colombia [3]. The presentation was complemented through a surgeon’s vision, i.e., with the participation from Dr Ricardo Oliveros, Head of Gastroenterology at Instituto Nacional de Cancerología in Colombia. He mentioned that 60% of cancers in the country are diagnosed in the advanced stages which makes the prognosis unfavourable. A Chilean colleague, Dr Christian Caglevic, from the Arturo López foundation in Santiago de Chile, reviewed the different therapeutic strategies from a clinical oncologist perspective.

Closing the session, Professor Antonio Lombard, from the Instituto Valenciano de Oncología of Spain, spoke about gastrointestinal stromal tumours (GISTs). His talk was about its management and how new treatments have revolutionised the natural evolution of these tumours.

Cervical cancer is a public health problem in developing countries. Nowadays it is a preventable illness. This is thanks to HPV vaccination and the study of the virus DNA which is more sensitive than the traditional Pap smear although more costly. In Colombia, both the vaccination and the DNA test are in the Obligatory Health Plan and are provided free of charge.

A few years ago, the vaccination programme against HPV suffered a setback because of a misinformation situation. It arose because of an adverse event that was attributed to HPV vaccination. It left many with a big question mark as to its nature and applicability. Because of this we invited Dr Nubia Muñoz, globally recognised for her fieldwork on the role of HPV and cervical cancer, to talk to us about the effectiveness and security of a tetravalent and nonavalent vaccine in the prevention of infection from HPV. Her dissertation was very clear and useful in dissipating uncertainties and outlining plans to reactivate the vaccination programme [4]. Dr Natacha Ortiz, consultant for the Liga Colombiana contra el Cancer, updated us on DNA tests of HPV for early detection of cervical cancer and the challenges one faces in clinical practice. To finish this module, Dr Javier Godoy, Head of Hospital Militar Central in Bogota, intervened with some comments about the futility of chemotherapy in controlling this illness. He emphasised the importance of strengthening advocacy and mitigation strategies. The breast cancer module was of great importance as this illness is the most frequent malignant tumour found in Colombia and the highest cause of mortality in middle age. He spoke of therapeutic advances and the economic implications involved. Dr José Caicedo, from Clinic del Country in Bogota, brought forward the idea of establishing an Oncology Centre in undergraduate programmes in Medicine Faculties. Prostate cancer, which is the most frequent malignant tumour in Colombian men, was extensively analysed by Argentinian colleagues who revealed new important therapeutic advances including robotic surgery and new technologies in radiotherapy.

Lung cancer was another focus for the congress. This topic aroused the keenest interest among all those addressed and high expectations from the speakers. Dr Luis Larrea from Hospital NISA, from Valencia in Spain, illustrated what can be done with radiosurgery and the modern techniques in the management of these tumours. Dr Danay Saavedra, immunologist from the Centro de Investigaciones Moleculares in Cuba, spoke to us about the therapeutic vaccine CIMAVax-EGF and the results taken over several years in non-small cell lung cancer patients. The results were very interesting and raised many concerns among attendees [5, 6]. Dr Mauricio Peláez, head of Servicio de Cirugía de Tórax de la Universidad Javeriana de Bogota, shared the point of view of a thorax surgeon in the management of bronchogenic cancer and emphasised transdisciplinary management of the disease.

The haematology module focused on multiple myeloma and the recent advances made in its diagnosis, treatment, and prognosis. This disease/illness was explained by Dr Rocío Orduz and Dr Myriam Rodríguez from the Fundación Santa Fe4 de Bogotá [7]. Another topic that sparked a lot of interest was on thrombosis associated with cancer. It is a fairly frequent complication seen over the years in practice at oncology clinics although its management has changed recently [8].

Lastly, an entire session was dedicated to palliative care with the conviction that this forms a fundamental pillar in supporting oncological patients. Dr Juan Guillermo Santacruz spoke about the use of cannabinoids for nausea and pain relief.

Another widely attended event was a workshop on radiotherapy advances. It was planned and developed by colleagues Beatriz and Marco Amendola from Miami, USA, in collaboration with other recognised radiotherapists from various countries. It was rated as excellent by 100% of the attendees.

## Conclusion

For the Liga Colombiana contra el Cancer, it was an extraordinary experience and a privilege to have organised the first Oncology Congress in Colombia with the *e*cancer Latin America team. We hope to repeat it in 2018.

## References

[1] Goss PE (2013). Planning cancer control in Latin America and the Caribbean. Lancet Oncol.

[2] Lichtman SM (2014). Geriatric oncology: an pverview. J Clin Oncol.

[3] Otero WR (2008). Cáncer Gástrico en Colombia. Rev Col Gastroenterol.

[4] Muñoz N (2010). Impact of human papillomavirus HPV vaccine on all HPV-associated genital diseases in young women. J Natl Cancer Inst.

[5] Vinageras EN (2008). Phase II randomized controlled trial of an epidermal growth factor vaccine in advanced NSCLC. J Clin Oncol.

[6] Keck CW (2016). The US and Cuba-turning enemies into partners for health. N Eng J Med.

[7] San Miguel JF Introduction to a series of reviews on multiple myeloma. Blood.

[8] Elyamany (2014). Cancer associated thrombosis: an overview. Clin Med Insights Oncol.

